# Primary intrathoracic malignant mesenchymal tumours: computed tomography features of a rare group of chest neoplasms

**DOI:** 10.1007/s13244-013-0306-0

**Published:** 2014-01-10

**Authors:** Marcel Koenigkam-Santos, Gregor Sommer, Michael Puderbach, Seyer Safi, Philipp Albert Schnabel, Hans-Ulrich Kauczor, Claus Peter Heussel

**Affiliations:** 1 Department of Diagnostic and Interventional Radiology, University of Heidelberg, Im Neuenheimer Feld 110, 69120 Heidelberg, Germany; 2 German Cancer Research Centre (Deutsches Krebsforschungszentrum—DKFZ), Im Neuenheimer Feld 280, 69120 Heidelberg, Germany; 3 Department of Radiology, University Hospital of the School of Medicine of Ribeirao Preto—University of Sao Paulo, Av. Bandeirantes 3900, Campus Universitario Monte Alegre, 14048 900 Ribeirao Preto, SP Brazil; 4 Clinic of Radiology and Nuclear Medicine, University Hospital Basel, Petersgraben 4, 4031 Basel, Switzerland; 5 Chest Clinic (Thoraxklinik), University of Heidelberg, Amalienstr. 5, 69126 Heidelberg, Germany; 6 Surgery Department, Chest Clinic (Thoraxklinik), University of Heidelberg, Amalienstr. 5, 69126 Heidelberg, Germany; 7 Institute of Pathology, Heidelberg University, Im Neuenheimer Feld 224, 69120 Heidelberg, Germany

**Keywords:** Neoplasm, Malignant mesenchymal tumour, Sarcoma, Thoracic cavity, Computed tomography

## Abstract

**Objectives:**

To describe the computed tomography (CT) features in a case series of primary intrathoracic extracardiac malignant mesenchymal tumours (sarcomas).

**Methods:**

A 5-year retrospective research was conducted, and 18 patients were selected. CT exams were reviewed by two chest radiologists, blinded to tumour pathological type, origin and grade. Lesions were described in relation to location, size, shape, margins, enhancement, presence of cavitation, calcifications, ground glass component, intratumoural enhanced vessels, pleural effusion, pleural tags, lymphangitis, chest wall/rib involvement and pathological lymph nodes.

**Results:**

The readers described five pulmonary, six mediastinal and seven pleural/wall based lesions. Mean largest diameter was 103 mm. The most frequent shape was irregular (*n* = 12), most predominant margin was smooth (*n* = 12) and enhancement was mostly heterogeneous (*n* = 8). Intratumoural vessels and pleural effusion were seen in 11 patients. Pathological lymph nodes were present in four cases and calcifications in two cases.

**Conclusions:**

Some frequent radiological features were described independently of tumour location and subtype. A sarcoma should be included as a major differential diagnosis when the radiologist faces an intrathoracic mass of large size (>70 mm) but with well defined smooth or lobulated margins, especially if presenting intratumoural vessels, associated pleural effusion but no significant lymphadenopathy.

***Main messages*:**

• *Malignant mesenchymal tumours (sarcomas) are rare and can arise from any structure in the chest.*

• *Intrathoracic sarcomas show some frequent radiological features, independent of location and type.*

• *Some CT features may help the radiologist suspect for a sarcoma instead of other more common tumours.*

## Introduction

Malignant mesenchymal tumours or sarcomas represent a diverse group of neoplasms that are of mesenchymal origin, have a malignant behaviour and are classified according to the tissue of origin and histological differentiation [1]. Thoracic primary sarcomas are rare neoplasms and they can arise from any structure in the thorax, including the chest wall, heart, pericardium, great vessels, lungs, pleura and breasts. Most commonly, sarcomatous lesions in the thorax represent metastases from extrathoracic musculoskeletal tumours, and when primary, characteristically they have evident origin in the chest wall or heart [2]. Predominant histological subtypes are also related to the site of origin. Chest wall tumours are mostly represented by the Ewing sarcoma, primitive neuroectodermal tumour (PNET), chondrosarcoma, osteosarcoma and liposarcoma [3]. In the heart, angiosarcomas represent the most common primary cardiac malignancy [4]. And when originating from mediastinal or pleuropulmonary structures, other subtypes become more prevalent, e.g. leiomyosarcoma, rhabdomyosarcoma, malignant fibrous histiocytoma, fibrosarcoma and synovial sarcoma. In these cases, when origin from the chest wall or cardiac/pericardial structures is not evident, sarcomas may mimic other more common thoracic neoplasms, including the bronchogenic carcinomas, mesothelioma, thymoma, lymphoma and germ cell tumours [5].

Imaging evaluation of thoracic sarcomas usually focuses on determining the extent of tumour involvement, potential for resectability and response to therapy [6]. Routine evaluation is initially done with conventional radiography and computed tomography (CT). For a better depiction of the chest wall soft tissue or mediastinal involvement, magnetic resonance imaging (MRI) can be performed [7]. In the evaluation of cardiac masses, cardiac MRI has also proved to be beneficial [8]. Despite of the advantages of MRI in characterization of chest wall and mediastinum masses, CT is still the most common imaging modality applied to the evaluation of intrathoracic masses in clinical routine. Even considering its most important role for disease staging, recent studies have showed that morphological characterization at CT may also help to suggest a tumour’s histological type and even add prognostic information. This has been markedly demonstrated, for example, for pulmonary adenocarcinomas [9]. Therefore, in this study, we aimed to describe the CT morphological patterns retrospectively assessed in a case series of malignant primary mesenchymal tumours located inside the thoracic cavity, not originating from the heart, that were evaluated in our institution and have confirmed histopathological diagnosis.

## Materials and methods

Retrospective search in the clinical database for thoracic malignant mesenchymal tumours evaluated at our hospital between 2007 and 2012 was conducted. First, all cases of metastatic disease, cardiac or pericardial tumours and lesions with clinical description of chest wall origin were excluded. Patients with sarcomatoid mesotheliomas were also not included in this study. Then, all patients who had pre-operative CT scans stored in the radiology archiving system were selected. Finally, patients with neo-adjuvant therapy prior to scanning and those with lesions described in the radiological reports as originating from or predominantly involving the chest wall were also excluded, resulting in a total of 18 patients selected for review. CT technique was variable, because exams were performed in different scans and some patients had pre-interventional exams performed in other centres but stored in the archiving system for comparison and treatment control. Medical records were reviewed and patient age, sex, treatment choice, follow-up and outcome was recorded.

All pre-operative CT exams were retrospectively reviewed by two chest radiologists who were unaware of the tumour histology subtype, origin and patient outcome. All lesions were confirmed to be intrathoracic and were consensually described in relation to their location (anterior or posterior mediastinum, lung, pleural/chest wall based), size (largest transversal diameter), shape (round/ovoid, irregular, undetermined), margins (smooth, lobulated, spiculated, undefined), enhancement (homogeneous, heterogeneous, with large necrosis), as for the presence of cavitation (gas within tumour), calcification, ground glass attenuation component, identifiable pathological intratumoural enhanced vessels (tortuous and dilated, different from normal pulmonary vasculature), associated pleural tags, pleural effusion, lymphangitis and chest wall (soft tissue) or rib involvement. Suspicious lymphadenopathy (short diameter >10 mm) and metastatic lesions in the chest scan (osseous, pulmonary, hepatic) were also recorded.

## Results

**Fig. 1:**
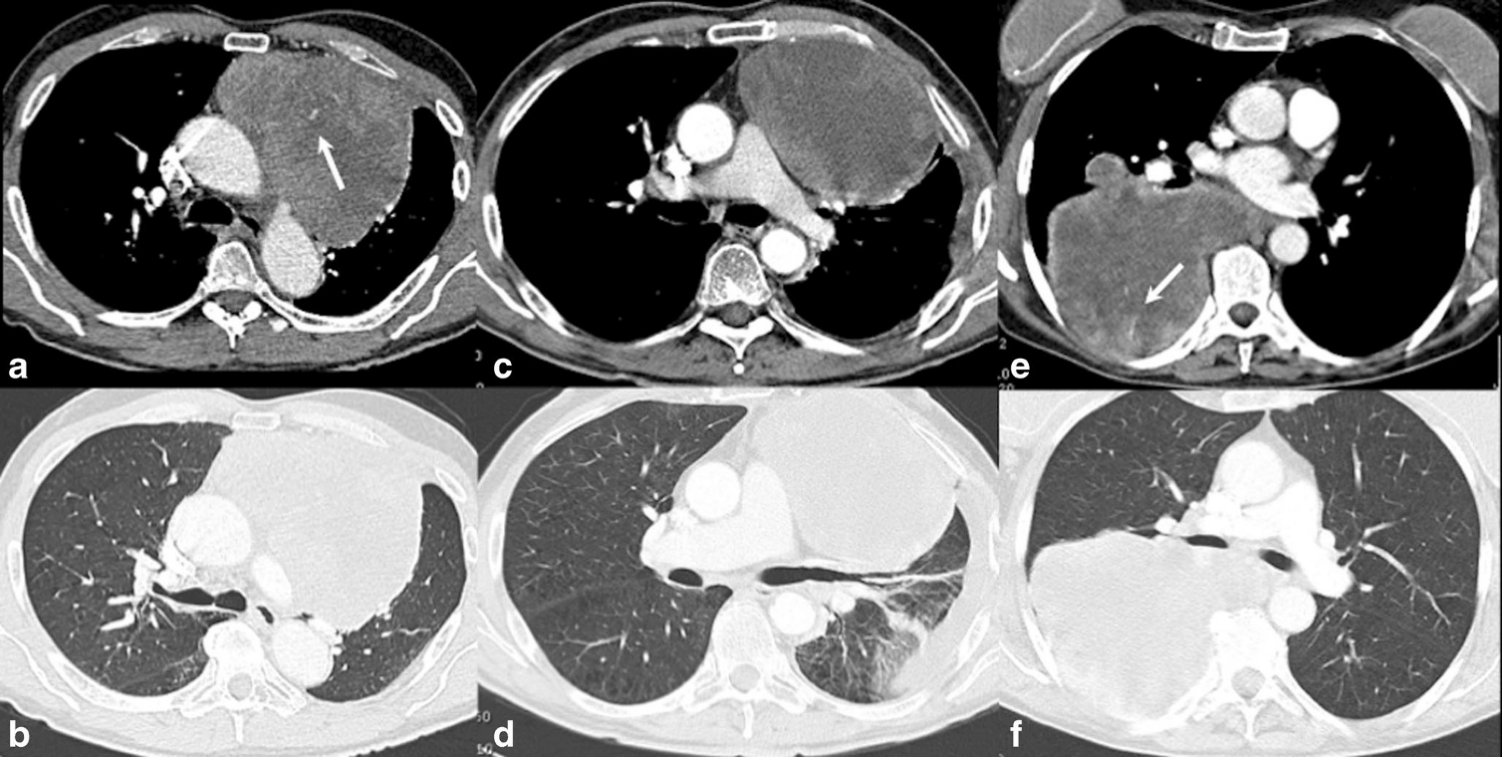
CT images of primary intrathoracic sarcomas. 63-year-old man (**a** and **b**) with undifferentiated sarcoma, presenting a large mass (143 mm) in anterior mediastinum, with ovoid shape, smooth margins, heterogeneous enhancement and intratumoural enhancing vessels (*arrow* in **a**). A 68-year-old man (**c** and **d**) with synovial sarcoma of the anterior mediastinum, showing ovoid shape, smooth margins, large necrosis and associated pleural effusion. A 67-year-old woman (**e** and **f**) with leiomyosarcoma of posterior mediastinum, mass with irregular shape, lobulated margins, necrosis and also intratumoural vessels (*arrow* in **e**)

**Fig. 2:**
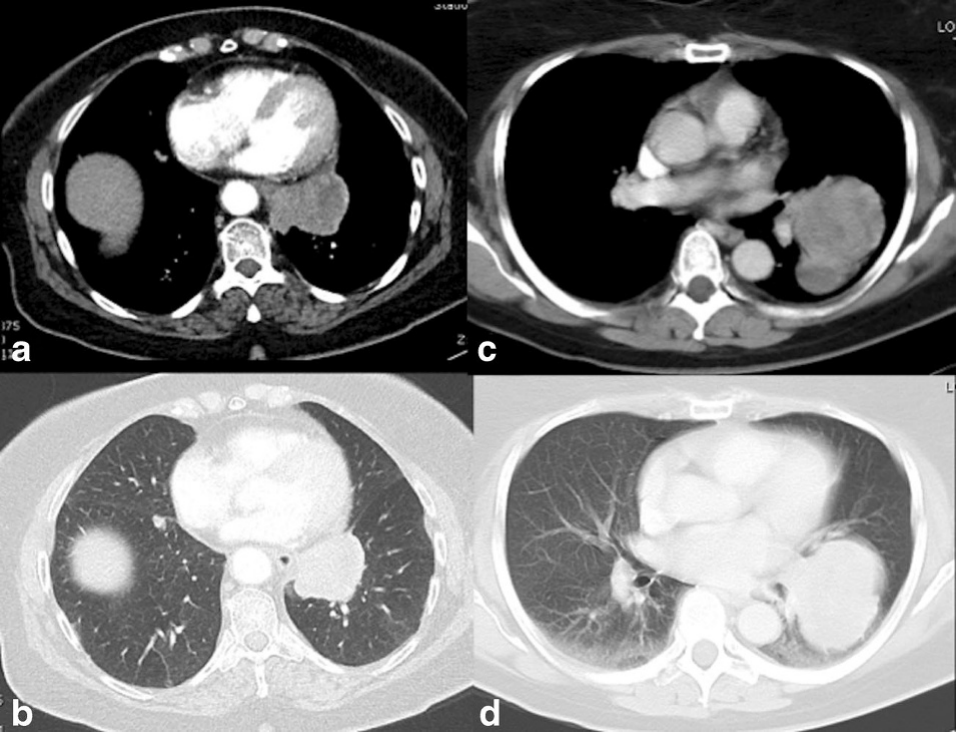
CT images of primary pulmonary sarcomas. A 67-year-old woman (**a** and **b**) with synovial sarcoma in the left lower lobe, presenting irregular shape, smooth margins and heterogeneous enhancement. A 61-year-old woman (**c** and **d**) with leiomyosarcoma, showing irregular shape, lobulated margins and heterogeneous enhancement

**Fig. 3:**
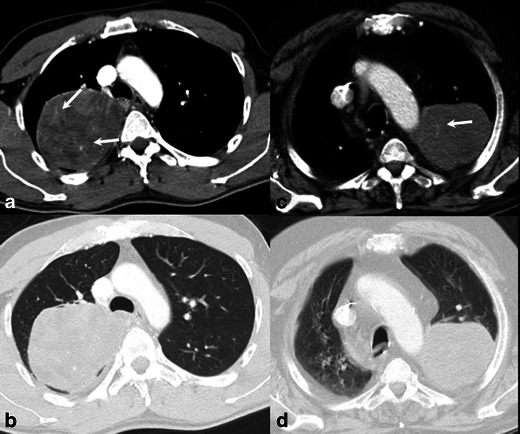
CT images of primary pulmonary sarcomas. A 39-year-old man (**a** and **b**) with synovial sarcoma in the right upper lobe, mass of ovoid shape, smooth margins, heterogeneous enhancement and prominent intratumoural vessels (*arrows* in **a**). A 86-year-old man (**c** and **d**) with angiosarcoma, also showing a large mass with smooth margins and identifiable intratumoural vessels (*arrow* in **c**)

**Fig. 4:**
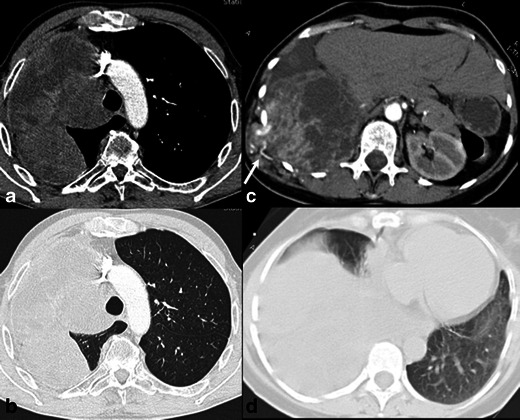
CT images of malignant solitary fibrous tumour. A 76-year-old man (**a** and **b**) with presenting as a large lesion, with irregular shape, lobulated margins and important necrosis. A 60-year-old woman (**c** and **d**) with malignant solitary fibrous tumour, with a mass presenting necrosis, intratumoural vessels, infiltration of the chest wall, and associated prominent collateral feeding vessels also seen outside the tumour (*arrow* in **c**)

**Fig. 5:**
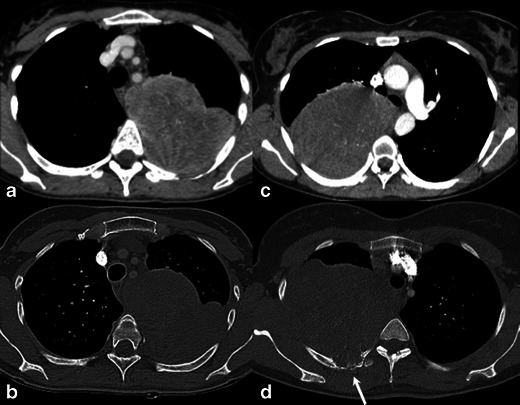
CT images of intrathoracic sarcomatous masses originating from chest wall. A 31-year-old woman (**a** and **b**) with Ewing sarcoma, a large mass with irregular shape, lobulated margins, heterogeneous enhancement, intratumoural vessels but no rib erosion. A 34-year-old woman (**c** and **d**) with a PNET, presenting an irregular mass, with smooth margins, heterogenous enhancement, rib erosion and intratumoural vessels—these showing a radial pattern in relation to the osseous destruction (*arrow* in **d**)

**Fig. 6:**
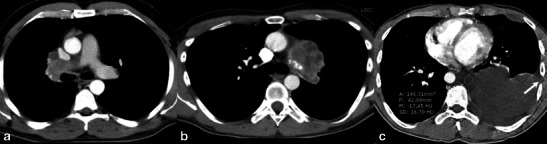
CT images of primary intrathoracic sarcomas. A 41-year-old man (**a**) with pulmonary artery undifferentiated sarcoma, lesion showing irregular shape and necrosis, with component that invades the lumen of the right pulmonary artery (intraluminal tumour). A 38-year-old man (**b**) with a mediastinal osteosarcoma, with irregular shape, lobulated margins, necrosis and intratumoural calcifications in the lesion’s medial aspect. A 26-year-old man (**c**) with liposarcoma, presenting a lesion of undetermined shape and undefined margins, infiltration of the chest wall (*arrow*) and components of low attenuation (ROI)

Among the 18 patients studied, 11 were men and 7 women (mean age, 55 years; range, 26-86 years). Fourteen patients were submitted to surgical resection of the tumour, while four were biopsied (three incisional and one percutaneous core-cut guided by CT). Table 1 summarises patients’ clinical and histological data. Figures 1, 2, 3, 4, 5 and 6 show images from all studied tumours. According to the surgical and pathological description, nine (50 %) tumours originated in mediastinum, four (22 %) in the lungs, three (17 %) in the pleura and two (11 %) in costal arch with lung infiltration. CT readings described five pulmonary, six mediastinal (five anterior and one posterior) and one pleural/wall based lesions. In six (33 %) cases the readers description of location differed from the surgical/pathological tumour origin. Two mediastinal tumours (patients 1 and 8) were radiologically described as located in the lung (central lesions), while another two (patients 4 and 7) were pleural/wall based lesions; one pleural tumour (patient 15) was described as located in the lung (peripheral lesion); and one pulmonary tumour (patient 10) was described as located in the posterior mediastinum.

**Table 1:** Clinical and pathological data of 18 primary intrathoracic malignant mesenchymal tumours

Patient	Gender	Age	Tumour type	Site of origin
1	Male	41	Sarcoma (undifferentiated)	Pulmonary artery (mediastinum)
2	Male	63	Sarcoma (undifferentiated)	Mediastinum
3	Male	68	Synovial sarcoma	Mediastinum (with lung invasion)
4	Female	67	Leiomyosarcoma	Mediastinum (with lung invasion)
5	Male	38	Osteosarcoma	Mediastinum
6	Male	54	Fibrosarcoma	Mediastinum
7	Female	52	Fibrosarcoma	Mediastinum (paravertebral)
8	Male	71	Synovial sarcoma	Mediastinum (with lung invasion)
9	Male	64	Sarcoma (undifferentiated)	Mediastinum
10	Female	67	Synovial sarcoma	Lung
11	Female	61	Leiomyosarcoma	Lung
12	Male	39	Synovial sarcoma	Lung
13	Male	86	Angiosarcoma	Lung
14	Male	26	Liposarcoma	Pleura
15	Male	76	Solitary fibrous tumour (with malignant features)	Pleura
16	Female	60	Solitary fibrous tumour (with malignant features)	Pleural (with chest wall invasion)
17	Female	31	Ewing sarcoma	Rib (with lung invasion)
18	Female	34	PNET (Ewing sarcoma family)	Rib (with lung invasion)

The mean largest tumour diameter measured at CT was 103 ± 33 mm. Only one tumour (pleural liposarcoma), which was not defined as a mass but as an infiltrative lesion of undetermined shape, could not be adequately measured. In 14 (78 %) cases the tumour measured >70 mm in maximum diameter and, therefore, according to the tumour-node-metastasis (TNM) staging system, would be classified as T3 tumours. Morphological features as described on the CT exams for all tumours are summarised in Table 2. The most frequent shape was irregular (12 cases, 67 %), the most predominant margin was smooth (12 cases, 67 %) and most common enhancement pattern was heterogeneous (eight lesions, 44 %). Round or ovoid shape was described in five patients (28 %). Calcifications were present in two (11 %, mediastinal osteosarcoma and mediastinal synovial sarcoma). Intratumoural vessels were seen in 11 tumours (61 %) and pleural effusion was also present in 11 (61 %) patients. Readers described tumours with rib or chest wall involvement in four patients (22 %), in the PNET, pleural liposarcoma, paravertebral fibrosarcoma and one solitary fibrous tumour (SFT). Presence of spiculated margins, cavitation, ground glass component, lymphangitis or pleural tags associated with the tumours were not described. Pathological thoracic lymph nodes were described only in four cases and pathologically confirmed in all. Suspected metastatic lesions identifiable in the chest CT were described in four patients and pathologically confirmed in two (liver metastasis in cases 8 and 9).

**Table 2:** CT morphological findings of 18 primary intrathoracic malignant mesenchymal tumours

CT findings	No. of tumours	Percentage
Location		
Mediastinum	6	33
Lung	5	28
Pleural/chest wall based	7	39
Shape		
Round/ovoid	5	28
Irregular	12	67
Undetermined	1	6
Margins		
Smooth	12	67
Lobulated	4	22
Spiculated	0	0
Undefined	2	11
Enhancement		
Homogeneous	3	17
Heterogeneous	8	44
Large necrosis	7	39
Calcification	2	11
Pleural effusion	11	61
Intratumoural vessels	11	61
Chest wall/rib involvement	4	22
Intrathoracic suspect lymph nodes	4	22

In some tumours, the readers also described other features. In one patient with a SFT (patient 16), tortuous and dilated enhancing vessels outside the tumour were present (aspect of collateral feeding vessels) (Fig. 4). In the patient with a PNET (patient 18), the large intrathoracic mass presented discrete adjacent rib erosion and some intratumoural enhanced vessels were described with a radial pattern in relation to it (Fig. 5). For the patient with pulmonary artery sarcoma, readers described the lesion with a component invading the lumen of the vessel (intraluminal solid tumour) (Fig. 6).

Mean follow-up time was 19 ± 18 months. Among the 14 patients undergoing surgical resection of the tumour, eight were treated with adjuvant chemo and/or radiotherapy. Two resected tumours were treated with neo-adjuvant chemotherapy prior to surgery and showed partial response (necrosis). The four patients with diagnosis based on biopsy were not submitted to surgery and treated with primary radio-chemotherapy, but all them evolved with progressive disease, and only one was still doing clinical follow-up in our hospital. Among all 18 patients, one (6 %) had a fatal outcome documented in our clinical records (tumour-related death, 4 months after surgery)—the patient with a resected mediastinal leiomyosarcoma. Ten (56 %) patients presented unfavourable evolution of the disease, with stable or progressive disease despite adopted treatment, and three among these patients were still being treated/followed in our hospital at the time of the last review of the records. One (6 %) patient with a pulmonary synovial sarcoma had a partial response to first sessions of adjuvant chemotherapy until the last control in our institution, and it was decided to continue with the therapy scheme. Six out of 18 patients (33 %) presented a favourable outcome, with complete response to therapy and no complications after surgery, and uneventful follow-up. The complete response group (cured patients) included the patients with Ewing sarcoma and PNET (Ewing sarcoma tumour family), as well as both patients with resected SFT.

## Discussion

Malignant mesenchymal tumours or sarcomas are rare tumours, and primary sarcomas of the chest are extremely uncommon, so that the diagnosis usually is reached only after sarcoma-like malignancies and metastatic disease have been excluded. Despite representing a small proportion of all thoracic malignancies, primary sarcomas of the chest are histologically represented by a large number and diverse group of lesions [10]. As regards the imaging features of these tumours, a review of the literature reveals limited information, consisting mainly of case reports and review articles [2, 3, 5, 6, 8]. Information becomes even scarcer when excluding the chest wall and the cardiac tumours. To our knowledge, there has been no previous report on the morphology features at CT of a series of intrathoracic and extracardiac primary sarcomas of different histological types and origins. In this study, we described 18 patients evaluated in a single institution over a time period of approximately 5 years, and despite the limited number of cases, common features were described that are helpful to suspect intrathoracic sarcomas.

Most of described cases in this series were represented by large masses (>70 mm). Despite their size, they still presented well-defined smooth or lobulated margins. Furthermore, round or ovoid shape, usually referred to as a sign of benign tumour, was described in one-third of the cases. Attenuation was heterogeneous or with large low-density areas suggestive of necrosis in most lesions, but cavitation was not found in any case. Pleural effusion was also a common feature, found in more than half of the cases. Suspicious lymphadenopathy was not frequent, described and pathologically confirmed only in four patients. Calcification was present in only one tumour. Other features like lymphangitis, pleural tags or ground glass component were not described in any case.

Similar descriptions of large heterogeneous masses but with well-defined margins were previously reported for different histological types of sarcomas inside the thoracic cavity. Zhang et al. [11] described the CT findings of five cases of primary pleuropulmonary synovial sarcomas, and all lesions presented as well-defined large masses with heterogeneous enhancement; associated pleural effusion was present in four patients and there was no lymphadenopathy and no tumoural calcifications. Pui et al. [12] described four patients with primary intrathoracic malignant mesenchymal tumours, and the typical imaging appearance was a large, well-circumscribed, non-cavitating, non-calcified mass without hilar or mediastinal lymphadenopathy. The feature of large tumours without significant lymphadenopathy was also described in a clinical study by Spraker et al. [13]. The authors reviewed the clinical characteristics and outcomes of 365 primary pulmonary sarcomas, 55 % of patients had large tumours (>5 cm) but only 16 % had node-positive disease. Eroglu et al. [14] reported a case of primary leiomyosarcoma of the anterior mediastinum presenting as a large (11-cm) mass without involvement of the main vessels or oesophagus, well-circumscribed, heterogeneous enhancement, associated pleural effusion but no lymphadenopathy. Cardinale et al. [15] described the CT features of 26 SFTs of the pleura with benign and malignant behaviour. The authors showed that up to 50 % of the lesions were larger than 10 cm, and even the largest lesions presented well–defined margins and lobulated contours, also with heterogeneous enhancement and important areas of necrosis, and 24 % of the lesions presented identifiable intralesional vessels.

In this study the presence of intratumoural vessels was a frequent finding, not only in SFTs, but also in other lesions (Figs. 1, 2 and 3). It is important to highlight the difference between the described intratumoural vessels and the positive angiogram sign. The angiogram sign represents the ability to see normal pulmonary vasculature within a parenchymal consolidation or opacity and was initially reported as specific for pulmonary adenocarcinoma (lepidic pattern) [16]. The intratumoural enhanced vessels are usually tortuous and dilated, and probably represent tumoural pathological feeding vessels. To our knowledge, this feature has not been described as characteristic for other mediastinal or pleuropulmonary tumours than SFTs [15, 17, 18].

One case of pulmonary artery sarcoma (undifferentiated type) was described (Fig. 6), showing a component that invaded the lumen of the right pulmonary artery (intraluminal tumour). Primary pulmonary artery malignancies are almost always sarcomas and comprise a variety of histological subtypes (malignant fibrous histiocytoma, angiosarcoma, leiomyosarcoma, fibrosarcoma, osteosarcoma and rhabdomyosarcoma). At imaging, the tumours typically present a central pulmonary artery filling defect associated with an intraluminal mass component that enhances with intravenous contrast material [19]. If evident expansion with extravascular extension or tissue enhancement is not present, pulmonary artery sarcomas may mimic thromboembolic disease, but usually adequate characterisation on CT or MRI is accurate to establish the diagnosis [20].

One Ewing sarcoma and one PNET were described in this series, in adult patients, aged 31 and 34 years, respectively (Fig. 5). Both lesions were radiologically described as intrathoracic pleural/wall based large masses, but only the PNET with associated rib erosion. Chest wall PNETs, also named Askin tumours, were previously classified as a separate group of neoplasms, but now are mostly accepted as an aggressive form of Ewing sarcoma, both representing the most common malignant tumours of the chest wall in children [3]. It was already described that this family of tumours, when originating from extraskeletal sites, occur in relatively older patients and may present as well-circumscribed, non-calcified masses, without the involvement of bone marrow (the hallmark of an osseous origin) [21]. Even though, finding of a large and heterogeneous mass with discrete rib destruction in a young patient is highly suspicious for a Ewing sarcoma family tumour [22]. Imaging findings are not sufficient to identify adjacent lung invasion.

One pleural liposarcoma was also described (Fig. 6). Approximately 10 % of the liposarcomas arise in the chest wall and most rarely from the pleura. The imaging appearance reflects the histological heterogeneity of this type of tumour, so that well-differentiated liposarcomas appear similar to fatty tissue and more poorly differentiated tumours have characteristics more similar to other sarcomatoid tumours. Presence of areas of low attenuation on CT or fat-typical signal intensity on MRI may suggest the diagnosis [3].

In conclusion, we described in this case series of 18 primary intrathoracic malignant mesenchymal tumours some frequent radiological features, independent of tumour location. These features are not characteristic for other more common neoplasms affecting the thoracic cavity, originating from pleuropulmonary (bronchogenic carcinomas, mesotheliomas) or mediastinal (thymoma, lymphoma) tissue. A sarcoma should be included as a major differential diagnosis when the radiologist faces an intrathoracic mass of large size (>70 mm) but with well-defined smooth or lobulated margins, especially if containing identifiable vessels inside the tumour, associated pleural effusion but no significant lymphadenopathy. Some tumours may even present more specific features, e.g. the intraluminal tumoural component of primary pulmonary artery sarcoma, a large mass with discrete rib erosion in a younger patient with Ewing sarcoma family tumour, and fatty tissue typical attenuation (on CT) or signal intensity (on MRI) of the liposarcoma.
